# Predictors of clinical recovery from vestibular neuritis: a prospective study

**DOI:** 10.1002/acn3.386

**Published:** 2017-03-22

**Authors:** Sian Cousins, Diego Kaski, Nicholas Cutfield, Qadeer Arshad, Hena Ahmad, Michael A. Gresty, Barry M. Seemungal, John Golding, Adolfo M. Bronstein

**Affiliations:** ^1^ Neuro‐otology UnitDivision of Brain SciencesImperial College LondonCharing Cross HospitalLondonUnited Kingdom; ^2^ NeurologyDunedin HospitalUniversity of OtagoDunedinNew Zealand; ^3^ Department of PsychologyUniversity of WestminsterLondonUnited Kingdom

## Abstract

We sought to identify predictors of symptomatic recovery in vestibular neuritis. Forty VN patients were prospectively studied in the acute phase (median = 2 days) and 32 in the recovery phase (median = 10 weeks) with vestibulo‐ocular reflex, vestibular‐perceptual, and visual dependence tests and psychological questionnaires. Clinical outcome was Dizziness Handicap Inventory score at recovery phase. Acute visual dependency and autonomic arousal predicted outcome. Worse recovery was associated with a combination of increased visual dependence, autonomic arousal, anxiety/depression, and fear of bodily sensations, but not with vestibular variables. Findings highlight the importance of early identification of abnormal visual dependency and concurrent anxiety.

## Introduction

Acute vertigo due to vestibular neuritis (VN), resolves over a matter of days but 30–50% of patients develop disabling chronic dizziness.^1^ Identifying predictors of the “acute‐to‐chronic” dizziness transition would allow patients at high risk of chronic dizziness to be targeted with focused therapies. Two possible predictors have been identified; follow‐up studies^2^, ^3^ have shown that psycho‐pathological features facilitate long‐term dizziness, however, cross‐sectional studies indicate that psycho‐physical estimates of how much an individual relies on vision for spatial orientation (“visual dependence”) is also associated with chronic dizziness.^4^, ^5^ As psychological questionnaires and psycho‐physical estimates of visuo‐vestibular interaction assess different functional domains, we now report a prospective study of VN patients examining visual dependence (rod‐and‐disk test), psychological features, as well as vestibulo‐reflex and vestibulo‐perceptual function. The aim is to establish how these variables interact to predict clinical outcome in VN.

## Method

Forty patients (mean age 50 years, range 22–79, 18 females) were studied prospectively in the acute phase of VN (1–5 days after onset, median = 2 days) and 32 patients in the recovery phase (median = 10 weeks). Twenty‐six of these patients were also seen in a long‐term recovery stage (median = 10 months) to validate acute and recovery stage findings. Acute clinical examination revealed unidirectional horizontal nystagmus with a torsional component, a positive horizontal head impulse test,^6^ unilateral caloric canal paresis, lateropulsion, and no hearing impairment or symptoms/signs of CNS disorder.^7^ Of the 24 patients who were administered prochloperazine, only three received these on the day of testing, but the drug has been shown to have no effect upon vestibulo‐reflex or vestibulo‐perceptual function.^8^ MRI brain scans were not routinely performed, but when done on hospital arrival (*n* = 3), no abnormalities were detected. No patient received corticosteroids. Patients were strongly advised to remain physically active and were explained the benefits of doing so but, in the acute phase, none were referred for formal physiotherapy.

In light of the aim of this study to assess how psychological variables interact with vestibulo‐reflex, vestibulo‐perceptual measures and visual dependency, at each stage patients underwent bithermal caloric testing (30–44°C) and the following test battery.

## Vestibular perceptual tasks

The **threshold vestibular task** (details in^9^) measures vestibular‐perceptual thresholds for detection of angular motion. The test comprised three rightward and three leftward rotations, with an initial acceleration of 0.5 deg/sec^2^, increasing by 0.5 deg/sec^2^ every 3 sec. Patients sat on a rotating chair with a hand‐held device with two buttons and were asked to press the button corresponding to their perceived direction of rotation (leftward/rightward) during each rotation. Vestibular‐perceptual thresholds were measured by the time taken from chair acceleration onset to button press.

The **suprathreshold vestibular task** (details in^9^) measures vestibulo‐perceptual responses to eight +/−90 deg/sec velocity steps lasting 60 sec with acceleration phase of 1 sec. Perceptual responses were recorded by patients turning a chair‐fixed tachometer wheel to indicate their perceived rotational velocity during the four rotational and four postrotational periods (starting‐stopping Barany test). The tachometer output follows an approximately exponential decay allowing measurement of the time constant of decay and the duration of the perceptual response.^9^ In this study, we only used the latter to reduce the number of variables statistically analyzed.

Rotations were performed using a vibration‐free motorized rotating chair (Contraves, USA; fitted with chin and head rests) in the dark with sound masking to eliminate nonvestibular cues.

## Rod‐and‐disk task^10^

**Figure 1:**
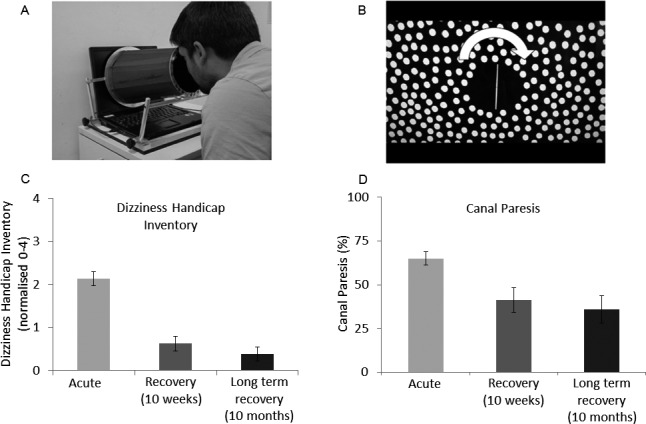
(A) Laptop‐based rod‐and‐disk test to measure visual dependency, showing a subject viewing the screen through a field‐restricting cone. Subjects carried out the test in a darkened room. (B) Laptop screen showing the randomly placed dots around the vertical line (rod) that subjects have to set up to vertical with a roller mouse (details in^5^). The task is carried out both with the background dots stationary and with dots rotating around the line of sight clockwise and counterclockwise (arrow) at 30 deg/sec. Visually induced rod tilt was used as a measure of visual dependence, calculated as the mean absolute rod tilt (in degrees) during disk rotation minus rod tilt values in the static condition.^4^ (C) Symptomatic recovery as measured by the DHI at the acute, recovery (10 week), and long‐term recovery (10 month) phases. DHI values are normalized from 0 to 4. Error bars are standard error of the mean. (D) Caloric canal paresis recovery. Most of the clinical (DHI) and caloric recovery takes place during the first 10 weeks. Despite this, individual clinical recovery is predicted by visual dependence rather than caloric improvement (see text). DHI, dizziness handicap inventory.

Visual dependence was measured with the rod‐and‐disk task on a laptop computer (Fig. 1A and B^5^; available at: http://www.imperial.ac.uk/medicine/dizzinessandvertigo ). Patients sat in front of the screen with the head held against an attached viewing cone to block extraneous visual cues. The stimulus consisted of a luminous white 6 cm rod against a black background filled with randomly distributed white dots. Patients had to align the rod to their perceived vertical (subjective visual vertical) with a roller mouse, from initial random rod settings ±40° from vertical, during four trials in three conditions: background dots stationary and dots rotating at 30 deg/sec clockwise and counterclockwise. Visually induced rod tilt was calculated as a measure of visual dependence for each subject. First, static tilt was calculated as the mean rod tilt in the four trials with background dots stationary. Then, visually induced rod tilt was calculated as the mean of the absolute values of the rod tilt from each trial with dots rotating minus the static rod tilt. Mean absolute static tilt was used as a measure of otolith function.

## Questionnaire measures

The **Dizziness Handicap Inventory** (DHI),^11^ measured the perceived handicapping effects of dizziness. A normalized score (0–4) was calculated by dividing total score by number of questions answered and used as an overall measure of recovery (0–1.3 = nil to mild handicap, 1.4–2.6 = moderate handicap and 2.7–4 = severe handicap). A normalized score was used as patients in the acute stage were not able to answer all questions, for example “Does walking down the aisle of a supermarket increase your problem?” The **Hospital Anxiety and Depression Scale** (HADS)^12^ measured state anxiety and depression. For each scale, scores ranged from 0 to 21 (high level of anxiety/depression). The **Body Sensations Questionnaire** (BSQ)^13^ measured the intensity of fear relating to body sensations, with scores ranging from 0 to 5 (extremely fearful). The **Vertigo Symptom Scale_arousal** (VSS_A)^14^ measured autonomic arousal components (e.g., heart pounding, excessive sweating; score 0–64).

## Analysis

The primary measure of outcome was symptomatic recovery as assessed with the Dizziness Handicap Inventory (DHI). Variables influencing symptoms at recovery (10 week_DHI) and long‐term recovery (10 month_DHI) stages were investigated using correlational (Pearsons ‘r’) analysis. We used stepwise multiple linear regression to predict outcome from baseline (acute) variables, whereas we used exploratory factor analysis^15^ to look for associations between significant variables and to assess whether these patterns of associations could be due to a small number of underlying factors (sometimes called unobserved or latent variables). Larzelere and Mulaik adjusted Bonferroni correction^16^ was used. Informed consent was obtained from all patients as approved by Charing Cross Hospital Ethics Committee.

## Results

Symptoms improved drastically from acute to recovery stages in all patients, with considerable individual variability (Fig. 1C). Average symptom load (Normalized DHI, score 0–4) decreased from 2.13SD1.02 acutely to 0.63SD0.95 at recovery stage (10 week). Results for all measures are summarized in Table S1.

### Predicting clinical recovery from the acute stage (Table 1)

**Table 1:** A matrix showing bivariate correlations between symptom recovery (DHI at 10 weeks) and acute psychophysical (threshold and suprathreshold tasks), canal paresis, visual dependency, and psychological variables

				Acute								
				Suprathreshold		Threshold						
		Age (years)	Canal Paresis (%)	Perception mean	Perception asymmetry (%)	Perception mean	Perception asymmetry (%)	Visual dependency	Static rod tilt	HADS	BSQ	VSS_A
DHI Recovery (10 Week)	Pearson Correlation	0.233	−0.239	−0.059	−0.172	0.066	0.057	**0.504**	0.252	0.176	**0.351** a	**0.529**
	Sig. (2‐tailed)	0.199	0.195	0.762	0.39	0.727	0.771	0.006	0.187	0.336	0.049	0.002

BSQ, body sensations questionnaire; HADS, hospital anxiety and depression scale. VSS_A, vertigo symptom scale_arousal

a Not significant after Larzelere and Mulaik adjusted Bonferroni test. 16 Bold indicates significance at the P>0.05 level.

DHI score at recovery stage (10 week _DHI) was significantly correlated with acute autonomic arousal (*r* = 0.53, *P* = 0.002), acute visual dependency (*r* = 0.5, *P* = 0.006), and acute fear of bodily sensations (*r* = 0.35, *P* = 0.049, not significant after Larzelere and Mulaik adjusted Bonferroni correction^16^). Stepwise multiple linear regression to predict DHI at recovery (10 week) stage, entering all the baseline acute variables is shown in Table 1 as predictors, produced a significant model (adjusted *R* ^2^=0.562, ANOVA *F* = 13.8, *df* 2,18, *P* < 0.001) in which the two significant predictors were acute autonomic arousal (*β* = 0.47 *P* = 0.02) and acute visual dependency (*β* = 0.41 *P* = 0.038).

### Associations between variables at the recovery stage

Table 2 shows all bivariate correlations between symptoms at the recovery stage (10 week) and psychophysical, visual dependency, and psychological variables also at 10 weeks. Clinical outcome (10 week _DHI) correlated with vestibulo‐perceptual thresholds (*r* = 0.52, *P* = 0.003), visual dependency (*r* = 0.56, *P* = 0.001) and, less significantly, with canal paresis (*r* = 0.38, *P* = 0.045, not significant after Larzelere and Mulaik adjusted Bonferroni correction^16^), all measured at 10 weeks. In addition, the following questionnaire data correlated with 10 Week_DHI: anxiety and depression (HADS, *r* = 0.71, *P* ≤ 0.001), autonomic arousal (VSS_A, *r* = 0.71, *P* < 0.001), and fear of bodily sensations (BSQ, *r* = 0.58, *P* = 0.001).

**Table 2:** Matrix showing bivariate correlations between symptom recovery (DHI at 10 weeks) and psychophysical (threshold and suprathreshold tasks), canal paresis, visual dependency, and psychological variables also measured at 10 weeks (recovery stage)

				Recovery (10 week)								
				Suprathreshold		Threshold						
		Age (years)	Canal Paresis (%)	Perception mean	Perception asymmetry (%)	Perception mean	Perception asymmetry (%)	Visual dependency	Static rod tilt	HADS	BSQ	VSS_A
DHI Recovery (10 week)	Pearson Correlation	0.233	**0.376** a	0.209	−0.093	**0.518**	−0.12	**0.556**	−0.115	**0.706**	**0.583**	**0.698**
	Sig. (2‐tailed)	0.199	0.045	0.268	0.626	0.003	0.519	0.001	0.532	<0.001	0.001	<0.001

BSQ, body sensations questionnaire; HADS, hospital anxiety and depression scale. VSS_A, vertigo symptom scale_arousal

a Not significant after Larzelere and Mulaik adjusted Bonferroni test. 16 Bold indicates significance at the P>0.05 level.

**Figure 2:**
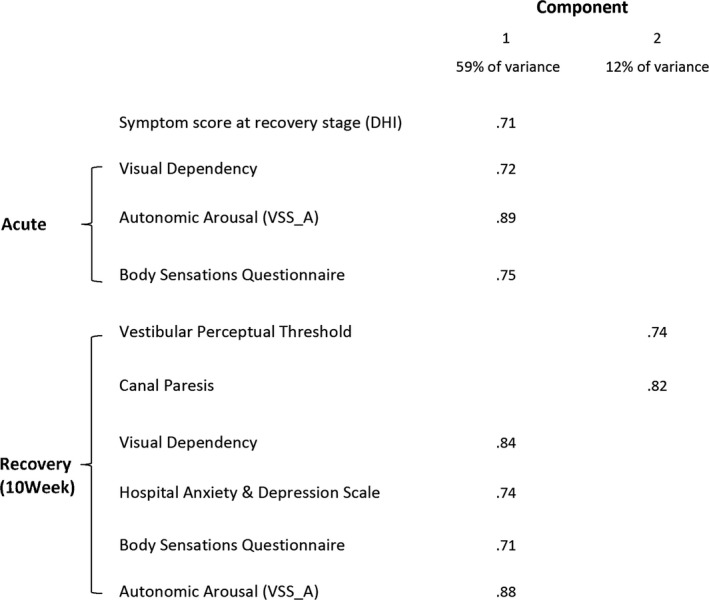
Factor analysis summarizing measures that correlate significantly (before adjustment) with symptomatic recovery (DHI at 10 weeks). Component 1 accounts for 59% of variance within the data set, and component 2 accounts for 12% of variance. For clarity those variables that load strongly (>0.7)^25^ on each component are only shown.

Factor Analysis (Fig. 2) was used to further explore the significant correlations outlined above and describe the underlying pattern of associations between variables. The first statistical component identified by Factor Analysis accounted for 59% of the variance and, critically, loaded 10 week_DHI, our outcome variable. This first component also loaded visual dependency, autonomic arousal, fear of body sensations (BSQ, acute and recovery stages), and anxiety‐depression scores (HADS, recovery stage). A second component was identified, accounting for just 12% of variance and loaded canal paresis and vestibular perceptual thresholds but, notably, did not include clinical outcome.

### Long‐term prognosis

Additional correlational analysis was carried out to assess whether factors determining symptomatic recovery outlined above, continue to predict symptom load at long‐term recovery (10 month_DHI). Comparisons between recovery (10 week) and long‐term recovery (10 month) stages showed no significant change in symptom load (DHI) (Fig. 1C), confirming that most recovery takes place by week 10. Bivariate correlational analysis showed acute autonomic arousal (VSS_A; r=0.78, *P* ≤ 0.001), visual dependency (*r* = 0.67, *P* = 0.001), and fear of bodily sensations (*r* = 0.46, *P* = 0.02) continue to predict long‐term outcome (10 month_DHI).

There was no significant difference in the baseline measures of autonomic arousal, visual dependency, or fear of bodily sensations between participants who returned at 10 weeks or 10 months and those who did not, suggesting drop‐outs did not systematically distort results.

## Discussion

We investigated how vestibular‐reflex (caloric), vestibulo‐perceptual, visual dependence (rod‐and‐disk), and psychological measures intertwine to predict clinical outcome in VN patients. Correlation and regression analyses showed that the main predictors of clinical recovery were increased levels of autonomic arousal (VSS_A) and visual dependence in the acute phase. Parameters in the recovery phase associated with clinical outcome were, again, visual dependency, anxiety/depression (HADS), autonomic arousal, and fear of bodily sensations. Vestibulo‐perceptual thresholds and, marginally, canal paresis at 10 weeks were also correlated with recovery. Critically, however, factor analysis revealed that visual dependency and questionnaire data loaded as a single factor, including the clinical outcome variable (10 week_DHI) and explained 59% of the variance. In contrast, the peripheral vestibular variables (caloric and threshold data) only accounted for 12% of the variance but, notably, did not include clinical outcome (Fig. 2).

Visual motion sensitivity and dizziness brought on by complex or moving visual surroundings are common in cross‐sectional studies of chronically symptomatic vestibular patients.^4^ Our prospective study shows that if too much weighting is placed on vision acutely (visual dependence), or if sensory integration mechanisms are unable to down‐regulate the visual contribution to the central compensation process, patients recover poorly. While prior studies have shown that anxiety, depression, and fear of body sensations are significantly associated with symptom recovery,^2^, ^3^ the novel finding is that it is the combination of psychological factors and visual dependence that best predicts clinical outcome. In agreement with previous studies, the degree of peripheral vestibular recovery (caloric, head‐impulse test or VEMPs) bears little influence on global clinical outcome.^17^, ^18^

Do autonomic arousal and psychological factors develop in response to heightened visual dependency, or vice‐versa, or are they coexisting independent parameters? The latter seems less likely given that compensation after a unilateral vestibular lesion relies upon multi‐sensory (visuo‐vestibular) reweighting, and central mechanisms subserving such functions are affected by psychological states.^19^ A mechanistic link between visuo‐vestibular compensation and psychological factors is underpinned by the presence of neuroanatomical networks processing visual, vestibular, and emotional inputs.^20^, ^21^. Moreover, fMRI data during simulated vertigo suggest an association between psychological traits and functional connectivity patterns within visuo‐vestibular and anxiety‐related cortical networks^22^, but the directionality of this association remains unclear. Our findings highlight (1) the importance of early identification of abnormal visual dependency and concurrent anxiety in VN and (2) the potential for early treatments to improve long‐term outcome by reducing visual dependency (sensory reweighting strategies^23^, ^24^) and combining pharmacotherapy and cognitive therapies to reduce anxiety and autonomic arousal. Further work should characterize the mechanism by which visual dependency is up‐regulated in such patients, in relation to increased anxiety, to allow more targeted therapies at the early phase of a vestibular injury.

## Author Contributions

SC was responsible for recruitment of participants and conducting testing, data analysis, statistical analysis, interpretation of data, manuscript development, and revisions. DK recruited participants, conducted testing and was involved with interpretation of data, manuscript development and revisions. NC recruited participants and conducted testing and was involved with interpretation of data and manuscript revisions. QA conducted participant testing and was involved with interpretation of data. HA recruited participants and conducted testing. MAG was involved in study concept and design, interpretation of data and study supervision. BMS recruited participants, and was involved in interpretation of data and critical revision of manuscript. JG conducted statistical analysis and was involved in revision of manuscript. AMB was responsible for study concept and design, interpretation of data, and was involved in all revisions of manuscript.

## Conflict of Interest

The authors declare no conflicts of interest.

## Supporting information


**Table S1.** Table summarizing results for all measures at acute, recovery, and long‐term recovery stages.
