# Supplementary material for: Predictors of clinical recovery from vestibular neuritis: a prospective study
Source: Ann Clin Transl Neurol. 2017 Mar 22;4(5):340–6. doi: 10.1002/acn3.386 (PMC5420806; doi:10.1002/acn3.386)
Supplement: Supplementary file 1 — Table S1. Table summarizing results for all measures at acute, recovery, and long‐term recovery stages. [file ACN3-4-340-s001.docx]

**Supplementary Table 1**

|  |  |  |  |  | Supra-threshold | | Threshold | |  |  |  |  |  |
| --- | --- | --- | --- | --- | --- | --- | --- | --- | --- | --- | --- | --- | --- |
|  |  |  |  |  |  | |  | |  |  |  |  |  |
|  |  | Age (years) | Canal Paresis (%) | Static rod tilt (degrees) | Perception Mean (seconds) | Perception asymmetry (%) | Perception Mean  (seconds) | Perception asymmetry (%) | Visual  Dependency (degrees) | HADS | BSQ | VSS_A | DHI |
| Acute | Mean | 49.65 | 65.03 | 4.75 | 21.17 | 1.59 | 15.84 | -8.99 | 8.01 | 10.41 | 2.41 | 10 | 2.13 |
|  | *SD* | *16* | *23.4* | *4.94* | *10.32* | *17.19* | *6.37* | *21.02* | *6.25* | *8.14* | *1.14* | *9.2* | *1.02* |
|  |  |  |  |  |  |  |  |  |  |  |  |  |  |
| Recovery (10Week) | Mean | - | 41.26 | 1.07 | 24.97 | 4.54 | 12.87 | -0.14 | 5.69 | 6.19 | 2.09 | 6.19 | 0.63 |
|  | *SD* | *-* | *37.99* | *1.31* | *12.93* | *11.73* | *6.02* | *15.12* | *4.57* | *6.59* | *0.97* | *8.38* | *0.95* |
|  |  |  |  |  |  |  |  |  |  |  |  |  |  |
| Long term recovery (10Month) | Mean | - | 36 | 0.90 | 24.05 | -4.63 | 11.40 | 3.93 | 6.23 | 4.67 | 1.83 | 5.38 | 0.38 |
|  | *SD* | *-* | *32.9* | *0.94* | *11.50* | *12.84* | *4.46* | *9.70* | *5.04* | *6.19* | *1.00* | *9.14* | *0.81* |
|  |  |  |  |  |  |  |  |  |  |  |  |  |  |
